# Leveraging social media data to study disease and treatment characteristics of Hodgkin’s lymphoma Using Natural Language Processing methods

**DOI:** 10.1371/journal.pdig.0000765

**Published:** 2025-03-19

**Authors:** Zasim Azhar Siddiqui, Maryam Pathan, Sabina Nduaguba, Traci LeMasters, Virginia G. Scott, Usha Sambamoorthi, Jay S. Patel

**Affiliations:** 1 Department of Pharmaceutical Systems and Policy, School of Pharmacy, West Virginia University, Morgantown, West Virginia, United States of America; 2 Real World Evidence, OPEN Health Evidence & Access, United States of America; 3 Department of Pharmacotherapy, College of Pharmacy, University of North Texas Health Sciences Center, Fort Worth, Texas, United States of America; 4 Department of Health Services Administration and Policy College of Public Health, Temple University, Philadelphia, Pennsylvania, United States of America; undefined, UNITED STATES OF AMERICA

## Abstract

**Background:**

The use of social media platforms in health research is increasing, yet their application in studying rare diseases is limited. Hodgkin’s lymphoma (HL) is a rare malignancy with a high incidence in young adults. This study evaluates the feasibility of using social media data to study the disease and treatment characteristics of HL.

**Methods:**

We utilized the X (formerly Twitter) API v2 developer portal to download posts (formerly tweets) from January 2010 to October 2022. Annotation guidelines were developed from literature and a manual review of limited posts was performed to identify the class and attributes (characteristics) of HL discussed on X, and create a gold standard dataset. This dataset was subsequently employed to train, test, and validate a Named Entity Recognition (NER) Natural Language Processing (NLP) application.

**Results:**

After data preparation, 80,811 posts were collected: 500 for annotation guideline development, 2,000 for NLP application development, and the remaining 78,311 for deploying the application. We identified nine classes related to HL, such as HL classification, etiopathology, stages and progression, and treatment. The treatment class and HL stages and progression were the most frequently discussed, with 20,013 (25.56%) posts mentioning HL’s treatments and 17,177 (21.93%) mentioning HL stages and progression. The model exhibited robust performance, achieving 86% accuracy and an 87% F1 score. The etiopathology class demonstrated excellent performance, with 93% accuracy and a 95% F1 score.

**Discussion:**

The NLP application displayed high efficacy in extracting and characterizing HL-related information from social media posts, as evidenced by the high F1 score. Nonetheless, the data presented limitations in distinguishing between patients, providers, and caregivers and in establishing the temporal relationships between classes and attributes. Further research is necessary to bridge these gaps.

**Conclusion:**

Our study demonstrated potential of using social media as a valuable preliminary research source for understanding the characteristics of rare diseases such as Hodgkin’s Lymphoma.

## Author Summary

This study explores the potential of using X (formerly Twitter) social media to study Hodgkin’s Lymphoma (HL), a rare cancer prevalent among young adults. By accessing posts from January 2010 to October 2022 through the X, we collected 80,811 posts to analyze disease-related discussions. We developed a Named Entity Recognition (NER), Natural Language Processing (NLP) tool to categorize posts into various HL-related topics, such as disease classification, progression stages, and treatments. The most commonly discussed topics in the posts were HL treatments and disease progression. The NER tool proved highly effective, with accuracy and F1 scores reaching up to 87% and 95%, respectively, demonstrating that social media can serve as a valuable platform for gathering preliminary data on rare diseases like HL. However, the study also recognized challenges in differentiating posts by patients, caregivers, or providers and pinpointing the timing of the discussed events, suggesting further improvement.

## Introduction

Social media platforms have been increasing in popularity among patients, caregivers, and providers for sharing health-related information with an online community, which provides rich and unique health data for research. [1–3] These platforms provide interesting observational real-world content in a non-clinical setting for digital phenotyping of patients, health surveillance, patients’ experiences, self-reported side effects, and patient’s complaints/and behaviors. [ 1,4–9] Despite the availability of various social media platforms, with Facebook as the most popular, strict privacy policies surrounding their data use make retrieving data from such platforms challenging. [10] In contrast, X (formerly Twitter) is a widely used, unrestrictive public platform offering extensive data and metadata from microblogging messages. [10,11] X provides real-time interaction through a brief message known as posts (formerly tweets), with a character limit for each post up to 280, increased from its historical limit of 140 characters on November 7, 2017. [12,13]

In 2021, a Pew Research survey of 1,502 U.S. adults evaluating social media use showed that 23% of participants reported using X. [14] Specifically, 25% of all men surveyed and 22% of all women surveyed stated they used the platform. [14] Furthermore, 42% of participants aged 18-29, and 7% of those aged 65 and older, reported using X. [14] Among the U.S. adults who stated they used the platform, 46% reported accessing it daily; additionally, the majority of the posts were replies (40%) or reposts (35%) of the original posts, indicating a wide distribution of messages. [13,14] The existing literature shows that X has been used for cancer-related research, with the majority of research on X focusing on highly prevalent cancers such as breast, prostate, and lung cancer. [15–18] For example, Sutton et al. evaluated lung cancer messages in X data and reported that most communication focused on treatment (32%), followed by end-of-life, prevention and risk information, and diagnosis, concluding that X is a valuable tool for health communicators and researchers in shaping cancer-related conversations and promoting awareness. [16] However, research on rare cancers with low incident rates is lacking.

Hodgkin’s lymphoma (HL) is a rare cancer of B lymphocytes characterized by the presence of Reed-Sternberg (RS) and Hodgkin (HRS) cells. [19–23] Despite its low incidence, accounting for 0.5% of all new cancer cases in the U.S., there were 223,512 survivors in 2020 due to an 88.9% five-year survival rate. [24] HL comprises classical HL (95% of cases) and nodular lymphocyte-predominant HL (NLPHL). [25] The incidence follows a bimodal age distribution, affecting individuals aged 20-30 and 65-75 years. [26–28] Risk factors include Epstein-Barr virus infection, familial predisposition, and immunocompromised status. [29,30] Common symptoms include asymptomatic lymphadenopathy, while advanced stages present with B symptoms such as fever, night sweats, and weight loss. [29,31] HL treatment outcomes are generally favorable, with most patients being cured through first-line combination chemotherapy with or without radiotherapy. [32–37] However, treatment options remain limited for relapsed or refractory cases, particularly after autologous stem cell transplantation.

As HL exhibits a high incidence rate in young adults, the demographics known for frequent use of X and the internet as a crucial source of obtaining health-related information, [38] it is likely that HL patients use X as one of the platforms to share their disease-related information and their experiences. Additionally, X has become popular among hematologists/oncologists for gathering and sharing clinical developments and trial discussions. [39,40] Given that oncologists primarily manage HL, this highlights the importance of social media platform X for studying HL. [41] Recent studies using natural language processing (NLP) to analyze cancer-related tweets have focused on sentiment analysis or a mixed-method approach that combines NLP-based sentiment analysis with qualitative content and thematic analysis of limited posts to study treatment and risk factors. [42–45] The literature lacks studies using X to explore HL, where unstructured social media data challenges traditional qualitative methods due to its volume and free-text nature. Consequently, a more efficient data-driven approach, such as natural language processing (NLP), which can automate the extraction of information from free text into structured data, is warranted.

Thus, the objective of this study is to assess the feasibility of using social media data to study disease and treatment characteristics of Hodgkin’s lymphoma using Natural Language Processing method.

## Materials and methods

### Overall workflow

A retrospective observational study using social media data employed the X API v2 developer portal, accessed through an academic research account (# 0263759948), to download posts from January 1, 2010, to October 31, 2022. Researchers can request access to X to download data for academic research. [46] We conducted a rule-based manual review of limited posts to filter out irrelevant posts. Irrelevant posts included those without relevant information on identified HL attributes, posts related to non-Hodgkin’s lymphoma, and false positives referencing “Hodgkin” in unrelated contexts.

**Fig 1:**
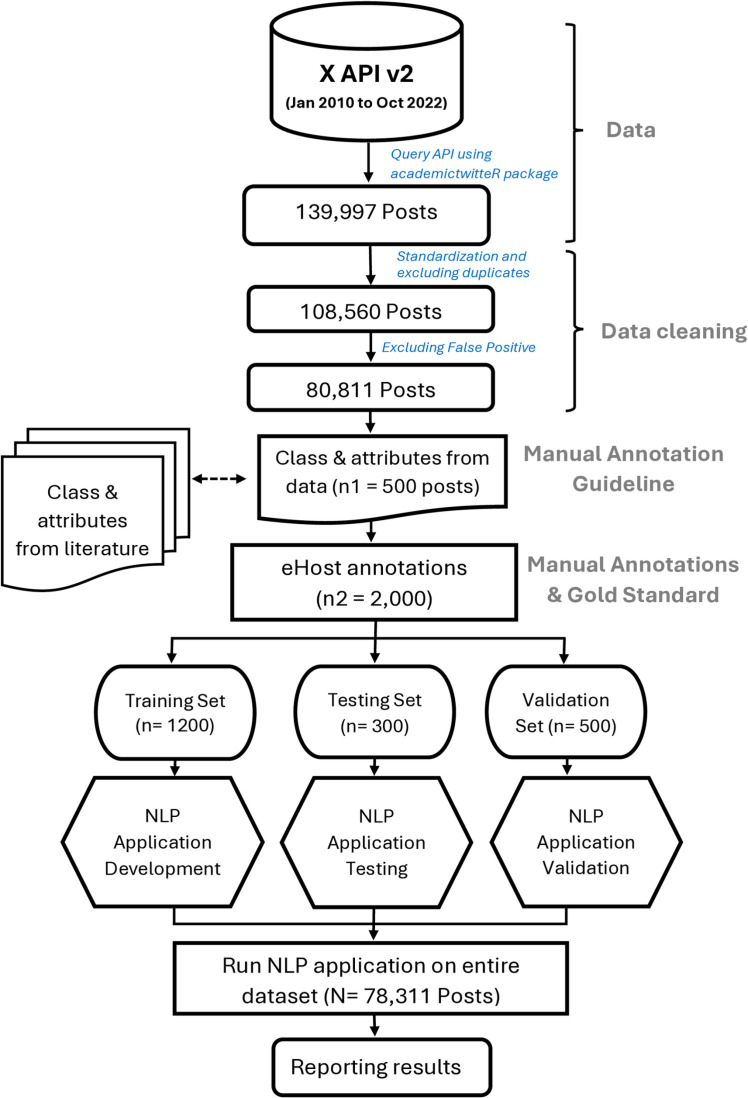
Overall workflow to develop a name entity recognition natural language processing (NLP) pipeline to extract Hodgkin’s lymphoma disease and treatment characteristics.

An annotation guideline was developed from the literature and manual review of 500 posts. This guideline was then utilized to create a gold standard dataset (2,000), divided into training, testing, and validation sets. The training dataset was used to develop the NLP application, and testing and validation were used to assess the performance of the NLP application. **Fig 1** shows the overall workflow of the methods.

### Data extraction and Preprocessing

We adopted an approach suggested by Kim et al. [10] to develop a search filter for “Hodgkin’s.” We utilized the R package- academictwitteR to query the X API. [47] The get_all_tweets() function was used to extract posts based on the inclusion and exclusion words list. The inclusion list comprised assorted lexical variants of “Hodgkin,” whereas the exclusion list consisted of “non-Hodgkin” variants. We incorporated “non-Hodgkin” and its variants in the query since the term contains the string “Hodgkin.” In addition, we excluded all reposts, promotions, media, images, and videos. Meanwhile, the reply, country, and post-specific geolocation data were captured whenever available. Posts location was determined based on the mapped location filter in X using the Profile Geo Enrichment algorithm.

The post-level data was extracted from API as a series of JSON files combined using the bind_tweets() function in the academictwitteR package. After the posts were extracted and combined, the data was standardized by converting posts into lowercase, removing usernames, non-numeric and non-alphabetic characters, emojis, entity objects, Uniform Resource Locator (URL), and non-English characters. After standardization and excluding duplicates and irrelevant posts, each post was converted into a separate text file that could be tracked with the unique ID.

***Inclusion words (lexical variants of Hodgkin):*** *“Hodgkin”, “Hodgkin’s”, “Hodgkin’s”, “Hodgkin”, “hodgkins”, “Hodgkins”, “#HodgkinLymphoma”, “Hodgkin”, “hodgkin”*

***Exclusion words (lexical variants of non-Hodgkin):*** *“non-hodgkin”, “non Hodgkin”, “Non-Hodgkin”, “non-hodgkin’s”, “non-Hodgkin”, “non-Hodgkin’s”*

### Annotation guidelines and gold standard dataset

We developed manual annotation guidelines to identify the concepts related to HL disease and treatment characteristics. We found a total of nine classes: HL classification, age-associated with HL, etiopathology, diagnosis and monitoring, site and region involvements, stages and progression, symptoms, disease and conditions, and treatment. These classes further diverged into attributes; for instance, class treatment included attributes- chemotherapy, immunotherapy, radiation therapy, stem cell transplants, and treatment aid. If a post is annotated into a particular class but does not fit into any designated attributes, it is considered a non-specific attribute for the specified class. For instance, posts mentioning “treatment received”, “medication”, and “novel therapies” were considered non-specific treatments as these treatments did not fit into any defined attributes for treatments. **Table 1** provides examples of manual annotation guidelines, while **S1 Table** displays mapped text for each class and its attributes, gathered during the manual review of posts and from literature during annotation guideline development.

**Table 1:** Manual annotation guidelines and examples.

Class & Attributes	Description	Posts examples
**HL classification:** Classic Hodgkin’s Lymphoma and Nodular lymphocyte-predominant Hodgkin lymphoma	It includes the classification of HL as Classical HL and its subtype (if mentioned): Nodular Sclerosis, Mixed Cellularity, Lymphocyte-rich, and Lymphocyte-depleted, and Nodular lymphocyte-predominant Hodgkin lymphoma	“finally got pathology report diagnosis stage **hodgkin nodular sclerosing lymphoma** treatable start therapy tonight thank support …..”
**Stages and progression:** Early stage (stages I & II), Advanced stage (stages III & IV), Remission, Cured, Recurrence/refractory, and decease	Stages and progression of HL. Stage I and II were combined as early, and III and IV as advanced. In addition, the disease progression as remission, relapsed, and recurrence were captured along with mortality due to HL.	“….. high school student received lifechanging **diagnosis stage iv** hodgkins lymphoma last april friday September…..,” “….. colleague suffering stage **hodgkin lymphoma relapsed** needs help pls come forward …..”
**Age associated with HL:** Pediatric, adolescent, and elderly	Age-associated with disease	“..… battle cancer **young age** diagnosed hodgkins lymphoma came shock..…”
**Etiopathology:** Epstein–Barr virus, familial risk/genetics, granulocyte-colony stimulating factor, PD-1 Inhibitors, Reed-Sternberg cells, and chimeric antigen receptors	Etiopatholgy and factors associated with increased HL risk	“….. today diagnosed ….. stage iv classic hodgkin lymphoma chl **reedsternberg** cancer.....” “….. youngest son ….. hodgkins disease **hereditary cancer** …..”
**Site and region involvements:** Neck/cervical, underarm, mediastinal, groin	Regions and sites that are commonly involved in lymph node	“….. wifes year old cousin started showing **lumps neck** weeks nd dose later turned hodgkin lymphoma..…”
**Diagnosis and monitoring:** Biopsy, MRI or CT scan, PET scan, X-rays	Biopsy and imaging used for the diagnosis and monitoring of HL	“ ….. surgeon perform **biopsy** excise suspicious lymph node weeks diagnosed hodgkins lymphoma today im still remission…..” “….. **pet scan** comes back …. went remission year ago stage hodgkins lymphoma couple weeks ago left armpit lymphnode swelled…..”
**Symptoms:** Lymphadenopathy, fever, sweats, weight loss, fatigue/tiredness, cough, shortness of breath, abdominal pain and/or swelling, and itchy skin	Symptoms commonly associated with HL. Lymphadenopathy (swollen) lymph nodes. Symptoms associated with B-symptoms: fever, drenching night sweats, and weight loss. In addition, other common signs and symptoms are presented in HL.	“…… **persistent itching cough** occurred ….. prescribed sleep aid xray later showed mediastinal mass trachea hodgkins lymphoma.….” “….. wife hodgkins **fatigue fever nightsweats wt loss dyspnea** finally saw different doc ……”
**Disease and conditions:** Digestive diseases, cardiovascular diseases, pulmonary diseases, mental health conditions, secondary cancers, infectious diseases, thyroid disorders, hair loss, and fertility issues	This class includes comorbidities and treatment complications such as secondary cancer, thyroid dysfunction, etc.	“…… feeling pretty shitty past days didnt get celebrate years remission today amp diagnosed stage hodgkins lymphoma **depression** hit really hard.…. eight years hodgkin disease ….. diagnosed **acute myeloid leukemia**”
**Treatment:** chemotherapy, immunotherapy, radiation therapy, stem cell transplants, treatment aid	Treatments associated with HL	“….. **brentauximab** injection wife suffering last stage hodgkin lymphoma nodular sclerosis gone refractory **first line cycle abvd**…..” “yo stage iv classical hodgkin lymphoma treated **brentuximab vedotin** bvavd x relapsed extranodal disease bsymptoms months completing **aavd** recommend salvage therapy”

Using annotation guidelines, two researchers, ZAS (PhD) and MP (PhD student), both with undergraduate degrees in clinical disciplines, independently annotated a subset of 100 posts using the extensible Human Oracle Suite of Tools (eHOST) annotation tool [48] to check their consensus. Interrater agreement (IRA) was performed to see the consensus between two annotators. Disagreement in the annotation, which included assigning class and attributes to the span of a posts containing pertinent information, was resolved by adjudication, resulting in a reference standard. IRA used to assess the consensus between two annotators was 93.2% (Cohen’s kappa), and for the individual class was: HL classification 98.1%, age-associated with HL 100%, etiopathology 100%, diagnosis and monitoring 94.7%, site and region 90.9%, stages and progression 89.2%, symptoms 68.4%, disease and conditions 84.2%, treatment 91.8%. Finally, a gold standard dataset was developed by manually annotating 2,000 posts, which was used to train, test, and validate the NLP application. eHOST saves annotated text in a structured XML Language. A bag of words and attributes was extracted for each class based on the annotation guideline and the mapped text class and attributes described in the S1 Table.

### Addressing false positive posts

During the manual review and annotation, we identified many posts extracted from X as False Positives (FPs). Although the query identified posts related to HL, the condition was actually absent. This occurred because the lymphoma is named after Dr. Thomas Hodgkin, which led to posts mentioning name “Hodgkin” getting identified as HL-related. To address this, we annotated posts where the string “Hodgkin” appeared but was not related to HL as FP. We developed a list containing FP “Hodgkin” words and excluded these from our data. **S2 Table** shows the list used for identifying and excluding FP posts.

### Developing a named entity recognition NLP application

The gold standard data set was split into a training set (n= 1200 (65%)), a testing set (n= 300 (15%)), and a validation set (n= 500 (25%)). The training dataset was used to develop a named entity recognition (NER) NLP application in Python to extract HL classes and attributes from posts automatically. The bag of words from the training dataset and the literature were used to train the application. Python library NLTK was used for essential NLP functions, such as tokenizing the sentences (posts) to generate the list of words from the sentence. [47] After tokenization, a lemma was used to create a panda data frame with each token and its associated lemma. Next, part of speech tagging and dependency parsing was performed to determine the relationship between each linguistic unit. Finally, the approximate string-matching algorithm (ASM) was used for name entity recognition NER NLP applications. ASM approximates the string search despite grammar and spelling errors using Levenshtein transpose distance algorithm for approximating string matching. [49] After developing the NLP application, we applied it to the entire dataset of 78,311 posts that were not used for developing applications. The tool systematically processed each post to identify and categorize relevant HL-related classes and attributes.

### Evaluating the performance of the NLP application

Application performance was evaluated using the validation dataset of gold standard data (n=500). For the validation dataset, we randomly selected an equal number of posts from each class, and the application outcomes for each class were compared against the manually annotated posts to determine the application’s performance using statistical measures such as precision, recall, accuracy, and F1 score.

## Results

We downloaded 139,997 posts between January 01, 2010, and October 31, 2022, using our inclusion/exclusion criteria. A minimal number of posts included the geo-coordinates (0.84%) and place ID (2.37%) required to ascertain the post’s country or geolocation. Restricting posts to these variables would significantly reduce the sample. Thus, we decided to include all posts irrespective of their geolocation data. After standardization, removing duplicates, and FP Hodgkin posts, the sample size was reduced to 80,811 posts. Of these, 500 were used for annotation guidelines, 2,000 for NLP application development, and 78,311 posts to apply developed applications.

**Fig 2:**
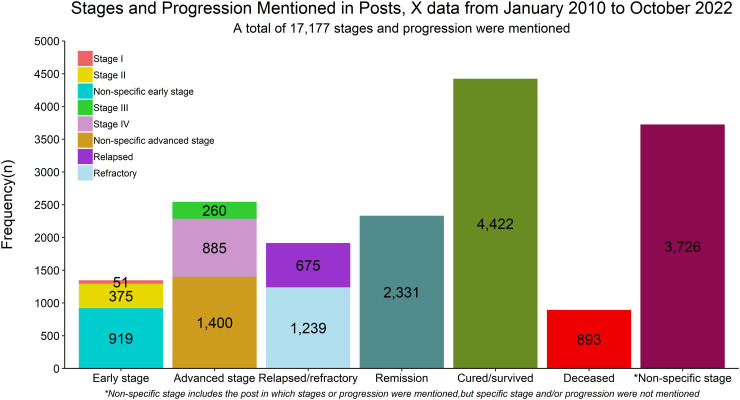
Different stages and progression reported in posts from January 2010 to October 2022 using X data.

In 3,340 posts, types of HL were mentioned, with 2,339 (70.03%) as classical HL and 341 (10.21%) as NLPHL. We found 17,177 (21.93%) posts that mentioned HL stages and progression. The majority of these posts, 4,422 (25.74%), were related to being completely cured, in which posts stated that the individual is “cancer-free” or has “beaten the cancer.” We combined stages I and II and non-specific early stages as early stages. Similarly, we combined stages III and IV along with non-specified advanced stages as advanced stages. Early stages accounted for 1,345 (7.83%), and advanced stages accounted for 2,545 (14.82%) posts. Remission was mentioned in 2,331 (13.57%), whereas relapsed/refractory was mentioned in 1,915 (11.15%) posts. **Fig 2** shows different stages and progression reported in the X data.

A total of 5,243 (6.67%) posts mentioned age, and the attributes included pediatrics 3,020 (57.6%), adolescents 1,298 (24.76%), and elderly 925 (17.64%). Class etiopathology was mentioned 1,200 times; in etiopathology, Reed-Sternberg cell 405 (33.75%) was most commonly mentioned, followed by Epstein-Barr virus involvement 360 (30%). Non-specific etiopathology was mentioned 149 (12.42%), and family involvement was mentioned 26 (2.17%) times. The chest (or mediastinal) region was the most commonly involved site 413 (39.6%), followed by the cervical region 326 (31.26%). In 283 (27.13%) posts, lymph node involvement was mentioned without mentioning specific sites of the body. Diagnosis and monitoring-related attributes were mentioned 2,646 times; in this class, non-specific diagnosis and monitoring method, in which no particular diagnosis means were mentioned 1,585(59.9%) times, followed by PET Scan 619 (23.4%) and biopsy 297 (11.2%).

We identified 865 specific events that constitute the symptoms of HL. Pain (236 (27.28%)), lymphadenopathy (178 (20.58%)), and fatigue/tiredness (156 (18.03%)) were the common HL-related; in addition, other symptoms include fever, itchy skin, sweat, cough, and weight loss. A total of 2,311 diseases and conditions co-occurring with HL were mentioned in the post, and the majority constituted secondary cancer 919 (39.77%). In addition to secondary conditions, lung diseases (365 (15.79%)), infection (352 (15.23%)), and cardiovascular diseases (304 (13.15%)) were commonly mentioned. Other conditions were related to mental health, abnormal blood counts, digestive problems, and hair loss.

**Fig 3:**
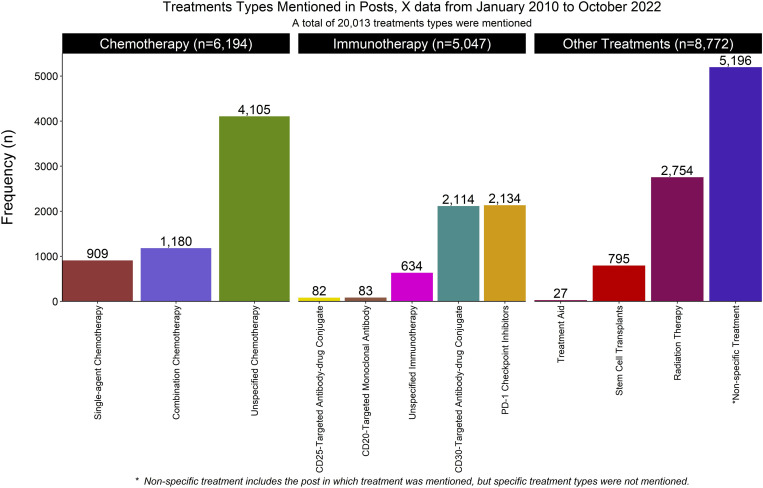
Types of treatment mentioned in Hodgkin’s lymphoma related posts from January 2010 to October 2022 using X data.

Treatment class received the most mentions, with 20,013 (25.56%) posts discussing HL-related treatments. Of these, chemotherapy was the most frequently mentioned treatment (6,194 (30.95%)), followed by non-specific treatments (5,196 (25.96%)) and immunotherapy (5,047 (25.22%)). Among the chemotherapy treatments, 1,180 (19.05%) were combination treatments, and 909 (14.68%) were single-agent chemotherapy. Within immunotherapy, Programmed Cell Death Protein 1 (PD-1) checkpoint inhibitors (2,134 (42.28)) were the most frequently mentioned agents. Additionally, CD30-targeting antibody-drug conjugates were the most commonly discussed agents in immunotherapy. **Fig 3** shows the treatment received; in the graph, the treatments are grouped into chemotherapy, immunotherapy, and all the other treatments combined, including non-specific treatment. **Table 3** shows the frequency of the extracted class and its attributes.

**Table 3:** Frequency of extracted class and its attributes.

Class	Attributes	Train (%)	Validation (%)	Entire dataset (%)
**Classification/types of HL**		**64 (100)**	**24 (100)**	**3,340 (100)**
	Classical Hodgkin’s lymphoma	32 (50.00)	11 (45.83)	2,339 (70.03)
	Nodular lymphocyte-predominant HL	7 (10.94)	11 (45.83)	341 (10.21)
	non-specific HL types	25 (39.06)	2 (8.33)	660 (19.76)
**Stages and progression**		**251 (100)**	**78 (100)**	**17,177 (100)**
	Early stage	20 (7.97)	2 (2.56)	1,345 (7.83)
	Advanced stage	30 (11.95)	8 (10.26)	2,545 (14.82)
	Recurrence/ refractory	28 (11.16)	8 (10.26)	1,915 (11.15)
	Remission	24 (9.56)	8 (10.26)	2,331 (13.57)
	Cured/ survived	56 (22.31)	21 (26.92)	4,422 (25.74)
	Decease	17 (6.77)	4 (5.13)	893 (5.20)
	Non-specific stages and progression	76 (30.28)	27 (34.62)	3,726 (21.69)
**Age associated with HL**		**75 (100)**	**39 (100)**	**5,243 (100)**
	Pediatric	32 (42.67)	20 (51.28)	3,020 (57.60)
	Adolescent	22 (29.33)	10 (25.64)	1,298 (24.76)
	Elderly	21 (28.00)	9 (23.08)	925 (17.64)
**Etiopathology**		**33 (100)**	**23 (100)**	**1,200 (100)**
	Reed-Sternberg cells	9 (27.27)	7 (30.43)	405 (33.75)
	Epstein–Barr virus	5 (15.15)	5 (21.74)	360 (30.00)
	PD-1 Inhibitors	5 (15.15)	2 (8.70)	236 (19.67)
	familial risk/genetics	3 (9.09)	2 (8.70)	26 (2.17)
	granulocyte-colony stimulating factor	3 (9.09)	2 (8.70)	15 (1.25)
	chimeric antigen receptors (CARs)	2 (6.06)	2 (8.70)	9 (0.75)
	non-specific etiopathology	6 (18.18)	3 (13.04)	149 (12.42)
**Site and region**		**24 (100)**	**15 (100)**	**1,043 (100)**
	Chest/mediastinal	8 (33.33)	5 (33.33)	413 (39.60)
	Cervical	7 (29.17)	3 (20.00)	326 (31.26)
	Lymph node	5 (20.83)	4 (26.67)	218 (20.90)
	Underarm	2 (8.33)	2 (13.33)	21 (2.01)
	non-specific site and region	2 (8.33)	1 (6.67)	65 (6.23)
**Diagnosis and monitoring**		**71 (100)**	**18 (100)**	**2,646 (100)**
	PET scan	15 (21.13)	4 (22.22)	619 (23.39)
	Biopsy	11 (15.49)	4 (22.22)	297 (11.22)
	X-rays	6 (8.45)	3 (16.67)	76 (2.87)
	MRI or CT	4 (5.63)	3 (16.67)	69 (2.61)
	non-specific diagnosis	35 (49.30)	4 (22.22)	1,585 (59.90)
**Symptoms**		**24 (100)**	**23 (100)**	**865 (100)**
	Pain	6 (25.00)	5 (21.74)	236 (27.28)
	Lymphadenopathy	4 (16.67)	4 (17.39)	178 (20.58)
	Fatigue/tiredness	2 (8.33)	3 (13.04)	156 (18.03)
	Fever	4 (16.67)	2 (8.70)	114 (13.18)
	Sweats	3 (12.50)	3 (13.04)	65 (7.51)
	Itchy skin	2 (8.33)	2 (8.70)	62 (7.17)
	Cough	1 (4.17)	2 (8.70)	43 (4.97)
	Weight loss	2 (8.33)	2 (8.70)	11 (1.27)
**Diseases and conditions**		**92 (100)**	**25 (100)**	**2,311 (100)**
	Secondary cancer	48 (52.17)	5 (20.00)	919 (39.77)
	Lung diseases	7 (7.61)	3 (12.00)	365 (15.79)
	Infection	7 (7.61)	3 (12.00)	352 (15.23)
	Cardiovascular disease	9 (9.78)	2 (8.00)	304 (13.15)
	Mental health conditions	4 (4.35)	3 (12.00)	91 (3.94)
	Abnormal blood counts	2 (2.17)	1 (4.00)	84 (3.63)
	Other diseases and conditions^*^	5 (5.43)	2 (8.00)	84 (3.63)
	Digestive problems	4 (4.35)	2 (8.00)	49 (2.12)
	Hair loss	2 (2.17)	2 (8.00)	38 (1.64)
	non-specific diseases and conditions	4 (4.35)	2 (8.00)	25 (1.08)
**Types of treatment**		**298 (100)**	**90 (100)**	**20,013 (100)**
	Chemotherapy	77 (25.84)	35 (38.89)	6,194 (30.95)
	Immunotherapy	55 (18.46)	14 (15.56)	5,047 (25.22)
	Radiation therapy	52 (17.45)	16 (17.78)	2,754 (13.76)
	Stem cell transplants	17 (5.70)	5 (5.56)	795 (3.97)
	Treatment aid	3 (1.01)	2 (2.22)	27 (0.13)
	Non-specific treatment	94 (31.54)	18 (20.00)	5,196 (25.96)

* Others includes hypothyroidism (n= 16), autoimmune diseases (n=10), fertility issues (n=9), etc.

### NLP performance

**Table 2** shows the performance of the NLP application on the 500 posts from the validation dataset of the gold standard. Overall, the concepts achieved 86% accuracy and 87% F1 score. Etiopathology performed best among all the classes, with 93% accuracy and 95% F1 score, followed by treatments and age associated with HL; both achieved an accuracy of 90% and an F1 score of 91%.

**Table 2:** Performance of the natural language processing application.

Measure	Overall	HL Types	Stages and progression	Age associated with HL	Etiopathology	Site and region involvements	Diagnosis and monitoring	Symptoms	Disease and conditions	Treatments
**Sensitivity/recall**	0.86	0.83	0.81	0.89	0.93	0.85	0.81	0.82	0.89	0.91
**Specificity**	0.85	0.86	0.88	0.92	0.95	0.81	0.95	0.86	0.64	0.90
**Precision/PPP**	0.87	0.89	0.89	0.92	0.97	0.81	0.96	0.85	0.67	0.91
**NPV**	0.84	0.78	0.79	0.88	0.86	0.84	0.76	0.83	0.88	0.90
**FPR**	0.15	0.14	0.12	0.08	0.05	0.19	0.05	0.14	0.36	0.10
**FDR**	0.13	0.11	0.11	0.08	0.03	0.19	0.04	0.15	0.33	0.09
**FNR**	0.14	0.17	0.19	0.11	0.08	0.15	0.19	0.18	0.11	0.09
**Accuracy**	0.86	0.84	0.84	0.90	0.93	0.83	0.87	0.84	0.75	0.90
**F1 Score**	0.87	0.86	0.85	0.91	0.95	0.83	0.88	0.84	0.76	0.91

PPV: Positive Predictive Value; NPV: Negative Predictive Value; FPR: False Positive Rate; FDR: False Discovery Rate; FNR: False Negative Rate.

Our application extracted the disease and conditions with the lowest accuracy of 75% and F1 score of 76%. While the recall for this class was 89%, the precision was only 67%. Upon error analysis of this class, we identified a high number of False Positive (FP) values, 20%, resulting in lower precision. A majority of the FP values in the class resulted from secondary cancer attributes. We identified three major themes behind the FP rates as below:

1. Post mentioned other cancers alongside Hodgkin’s lymphoma when no secondary cancer was present.

*“husband hodgkins aggressive nonhodgkins chemo received ……… registered government site definitely qualify one given priority slots except......”*

2. Post discussing cancers in two separate individuals

*“made years without knowing anyone cancer …….. slapped dad stage pancreatic cancer friend stage hodgkins lymphoma....”*

*“…….. cancer around present friends breast cancer hodgkin lymphoma plus hubby lung cancer ………………. much respite love carries forward strength”*

3. Differential diagnosis or instance of misdiagnosis

*“main stage hodgkins lymphoma almost died kept saying pneumonia tried send home refused demanded tests thats found listen ur body dont let doctors send u home without adequate answers…….”*

## Discussion

This study uses NLP to characterize the discussion of HL on the X platform. This approach represents a seldom-examined application of NLP in analyzing user-generated content to gain insights into a rare cancer, such as HL. Our primary finding shows excellent performance of the NER NLP application in extracting and characterizing HL-related information from posts, as indicated by a high F1 score for the overall application and the analysis of individual disease and treatment-related classes. Our results aligned with the existing literature on etiopathology NLP application performance. In addition, the frequency of attributes within classes, such as age, types of HL, and treatments, concur with the existing literature, further highlighting the relevance of our approach in extracting meaningful information from social media for preliminary research into rare diseases like HL.

Our NLP application achieved high performance, with etiopathology concepts outperforming others, with a 95% F1 score. These findings align with the study by Luo et al. [50] The authors used sentence subgraphs from the narrative pathology reports to classify HL and compared the system performance against the actual pathological report, achieving a high performance for the HL with a to assess the system performance F1 score of 0.91. [50] Authors concluded that distinctive pathological characteristics of HL, such as the presence of Reed-Sternberg cells, benefit in differentiating HL from other lymphomas and diseases and aid in identifying HL with high recall and precision. Additionally, other pathological features include specialized scientific terms such as PD-1 inhibitors and chimeric antigen receptors (CARs), which help NER achieve high classification performance due to the distinctiveness of such terms, thus enhancing the performance of the etiopathology class. [51] While Reed-Sternberg cells are a hallmark of HL diagnosis, the disease pathology can exhibit a complex microenvironment, with PD-1 inhibitors and CAR receptors associated with multiple cancers, making diagnosis challenging if solely based on these attributes. [52–54] Therefore, we believe associating pathological attributes with the required diagnostic term “Hodgkin” facilitated the excellent performance of the etiopathology class.

In contrast to the high-performing etiopathology class, the disease/condition class, though adequate, was the lowest performing among other classes, with an F1 score of 76%, indicating room for further improvement, especially in precision, with a score of 67%. The high recall rate indicated that the system could identify instances of diseases/conditions in the posts. However, the low precision due to the significant number of False Positives (FP) highlights the challenges in distinguishing actual instances of diseases/conditions from nuanced or irrelevant expressions of disease. Further investigation into the reasons behind FPs revealed three primary themes, denoting the complexity of language usage on social media platforms. For instance, the first FP theme involves health-related discussion where multiple conditions are mentioned together, even when not all conditions are present. This leads the NER system to falsely identify these as relevant diseases/conditions (secondary non-Hodgkin cancer in the given example). In the second FP scenario, the challenge lies in the complexity of assigning multiple attributes and subjects correctly, especially when the narrators jump between the subjects. Thus, the application cannot accurately assign the relevant entities to their class, affecting its precision and underlining the importance of contextual use of language.

The contextual use of language is highly relevant in our study, as we have data from different periods with varying grammar and linguistic structures introduced in the post after the X expanded its characters limits from 140 to 280 in November 2017. [12,13] Our study period extends from January 2010 to October 2022, providing a mix of posts before and after the character limit change. Previous studies have highlighted the impact of character limit change on the language used on the X platform; for instance, with the relaxed character limit, the use of articles, conjunctions, prepositions, and more formal language has been increased. [51] These changes introduce different grammar and linguistic patterns that can impact system performance. Therefore, training the data on a diverse set of pre-and-post character limit posts is crucial to ensure the robustness of the application against varying grammar and language structures, as we have done in our study. The changes in the character can impact both the precision and recall of the NER application. Longer posts have the potential to improve recall by introducing more entities but, at the same time, compromise precision if the application fails to recognize or correctly assign the entities within the increasingly complex linguistic structures.

Our findings show that 70.03% of posts mention classical Hodgkin lymphoma, 10.21% mention NLPHL, and the remainder were non-specified. This aligns with real-world distributions of HL subtypes, where classical HL accounts for approximately 95% and NLPHL the remainder. [25] The highest percentage of posts focused on remission or cure (39.31%), aligning with the excellent prognosis generally associated with Hodgkin lymphoma. [24] However, mentions of early stage diagnosis were low (7.83%), while advanced stages were more frequent (14.82%), contrasting with real-world data where early stage diagnosis is more common (60-70%) due to symptomatic presentations like swollen lymph nodes. [55,56] Among posts that specified age (6.67%), a high percentage (57.6%) concerned pediatric cases, contrasting with the actual age distribution of HL, which more commonly affects individuals aged 20-30 and 65-75. [26–28] This discrepancy could partly be attributed to the lower prevalence of social media use among individuals aged 65 and above. [14] Additionally, the tendency to share posts involving children with illness could be influenced by raising awareness, empathy, and social media algorithms, which prioritize emotionally engaging content, including those that evoke empathy. [57–59]

Treatment was the most commonly discussed topic in the posts, with 20,013 (25.56%) of the 78,311 posts on which the developed NER application was deployed. Among the specified treatments, combined chemotherapy (e.g., ABVD, AAVD, BEACOPP) followed by targeted immunotherapy and radiation therapy frequently occurred in the posts, as shown in Fig 3. Our findings on the treatments for HL align with the literature, which indicates that combination chemotherapy with or without radiation therapy is the first line of treatment. [34,35] In addition, the literature also shows that in advanced stages, chemotherapies are often combined with immunotherapies. [35,60] For instance, patients who relapse after autologous stem cell transplant (ASCT) or who are not eligible for ASCT are typically treated with targeted agents, such as CD30, which is selectively expressed in cHL. [61,62] Therefore, targeted agents, such as CD30-antibody drug conjugates (brentuximab vedotin) and PD-1 checkpoint inhibitors, are increasingly being used in combination with chemotherapy, and they were the most commonly mentioned immunotherapy agents in our posts.

An NLP application leveraging social media platforms like X can facilitate real-time health surveillance, enabling researchers to monitor large scale health discussions, patient-reported experiences, and public sentiment on treatments and disease outbreaks. For instance, Keim-Malpass et al. [63] analyzed HPV vaccine perceptions on X, finding a 51% polarized optimistic viewpoint, while Boucher et al. [64] observed that vaccine-supportive groups shifted focus from HPV during the COVID-19 pandemic. Additionally, X data is valuable for monitoring non-communicable diseases; for example, Eichstaedt et al. [65] showed that language patterns reflecting traits like anger and disengagement could predict atherosclerotic heart disease mortality, outperforming models based on traditional demographic and health factors. Hswen et al. [66] demonstrated how X data could aid in diagnosing autism spectrum disorder (ASD) through digital phenotyping, while platforms like PatientsLikeMe have shown that patient-generated data enhances disease self-management by enabling patients with conditions like epilepsy to connect and share insights. [67] Using NLP, researchers could apply a similar approach to X for identifying specific populations by analyzing language patterns associated with various conditions, which may reveal valuable insights into symptoms, treatment effects, and quality of life. Digital phenotyping through X data, as demonstrated by De Choudhury et al., [68] who used NLP to assess depression risk before onset, suggests that our application could similarly help researchers identify HL patients based on their treatment experiences and engagement with disease-related discussions, providing early insights into patient needs and supporting informed healthcare interventions.

Based on our understanding of the existing literature, this is the first study to leverage social media data using the NLP method to study HL patients’ disease and treatment characteristics. Our NLP application performed excellently, as evidenced by the F1 score for the overall application assessment and the assessment of individual disease and treatment-related classes. Our results suggest that X has the potential to offer an initial insight into rare diseases like HL.

Our study has several limitations, a major one being that it does not distinguish between posts written by patients, providers, and caregivers (patients’ families/relatives). Attributes that differentiate patients and caregivers were not sufficiently mentioned, as evident in our manual review. X is more prevalent among young individuals, as evidenced by our findings and literature; however, only 6.7% of the total posts in our analysis mentioned age-related attributes. Thus, it is highly likely that the posts will be skewed toward younger people, but we cannot assert this with certainty. We could not establish relationships and temporality between different classes and attributes because this information was missing from the posts, likely due to the character limit restrictions of the X platform. Finally, we might have missed some posts that included both false positive terms and true references to Hodgkin’s disease, during the API querying process.

In addition to the limitations of our study, broader challenges and ethical considerations arise when using social media data for research. Several studies have highlighted the potential risks of inadvertently identifying users from social media posts, threatening the confidentiality of identifiable information. [69–71] For instance, Ayers et al. found that 61% (68/112) of studies analyzing Twitter data could potentially reverse-identify at least one account holder, with 21% even disclosing usernames. [69] This underscores that, while social media users may not fully understand the extent to which their sensitive or identifiable information may be used in research, researchers must assume greater responsibility for anonymizing data and adhering to strict privacy standards, such as those outlined by the International Committee of Medical Journal Editors. These standards require that identifying information should not be published unless it is scientifically necessary, and in such cases, informed consent is required. [72] To strengthen ethical standards in social media based research, future studies should engage users through transparent outreach and obtain informed consent when necessary. Additionally, using aggregated data and exercising caution with identifiers, such as usernames, can ensure privacy and increase user trust in social media research.

Finally, future studies will require comparing our NLP application’s performance with other open-source NLP tools and validating our tools on other social media data. Further study is also needed involving other social media platforms and digital platforms of patients and caregiver communities, such as PatientsLikeMe and CaringBridge, with richer resources and the possibility of establishing a temporal relationship. Temporal attributes would help us understand the timeframe between disease diagnosis and outcomes, such as remission or progression, as well as other similar timeframes, including the administration of chemotherapy and the emergence of its associated side effects.

## Conclusion

This study highlights the untapped potential of social media as a preliminary research source in understanding the characteristics of rare diseases like Hodgkin’s Lymphoma using Natural Language Processing methods, as we demonstrated through our excellent application performance. Due to data constraints, we encountered a limitation in distinguishing between posts from patients, providers, and caregivers and in establishing temporal relationships between attributes. We proposed further research to validate our NLP application by comparing it with the existing application and testing it on additional data sources. Further continuous adaptation and evaluation of the application will be necessary to account for the evolving online X user community and the contextual use of language on such platforms.

## Supporting information

S1 Table Mapped text from each class and its attributes. (DOCX)

S2 Table List of words used to identify and exclude False Positive posts. (DOCX)
