# Supplementary material for: Leveraging social media data to study disease and treatment characteristics of Hodgkin’s lymphoma Using Natural Language Processing methods
Source: PLOS Digit Health. 2025 Mar 19;4(3):e0000765. doi: 10.1371/journal.pdig.0000765 (PMC11922232; doi:10.1371/journal.pdig.0000765)
Supplement: S1 Table — (DOCX) [file pdig.0000765.s001.docx]

**S1 Table: Mapped text from each class and its attributes**

| **Class** | **Attributes** | **Mapped text matches** |
| --- | --- | --- |
| Classification/types of HL | Classic Hodgkin's Lymphoma | classical HL, classical hodgkin, nodular sclerosing hodgkin |
|  | Nodular Lymphocyte-Predominant Hodgkin Lymphoma | lymphocytepredominant hodgkin lymphoma, nlphl, nodular lymphocyte predominant hodgkin lymphoma |
| Stages and Progression | Early stage | Class ii, Early hodgkin lymphoma, Early stage, Early stage unfavourable hodgkin lymphoma, Earlystage favorable hodgkin lymphoma, Hodgkin ii, Hodgkins lymphoma second grade, Stage iia, Stagei, Stage ii, Stage ii hodgkins disease, Stage ii hodgkins lymphoma |
|  | Advance Stage | Advanced, Advanced chl, Advanced classical hodgkin lymphoma, Advanced hodgkin, Advanced hodgkins disease, Advanced hodgkins lymphoma, Advanced stage, Advanced stage hodgkin lymphoma, Advanced-stage, Advances hodgkin lymphoma, Stage 4, Stage four, Stage four hodgkin, Stage iii, Stage iiia hodgkin lymphoma, Stage iiiiv, Stage iv, Stage iv , Stage-4, Stages iii hodgkin lymphoma, Terminal hodgkin lymphoma |
|  | remission/ recovering | Recovering, Remission, Shrinks |
|  | cured | Beat hodgkins lymphoma, Beating cancer, Cancer free, Cancer survivor, Cured, Free cancer, Full healing, Fully recovered, Healed hodgkins disease, Hodgkin lymphoma survivors, Survived, Healed |
|  | recurrence/refractory | Early relapse, Late relapse, Recurrence, Refractory, Refractory relapsed, Relapse, Relapsed or refractory, rr hodgkin lymphoma, Unresponsive |
|  | deceased | Dead, Decease, Died, Died hodgkins disease, Dying, Mortality, Passed away, Treatmentrelated mortality |
| Age groups | Pediatric | ChildhoodCancer, children, childhood hodgkins lympho, pediatric, pediatric hodgkins, paediatric patients, kids |
|  | Adolescent | Young, young adults, Teen, young women, young, adult, adult patients, young patients, young adults, adolescents |
|  | Elderly | old, older patients, elderly |
| Etiopathology | Epstein–Barr virus | EBV, EBV viral association, EBV-associated, viral association, ebv reactivation, epsteinbarr virus, subtypes epsteinbarr |
|  | familial risk/genetics | Familial clustering, genetic mutation, genetic variation, genetic abnormality, hereditary, genetics, familial risk |
|  | Reed-Sternberg cells | RS cells, rs cells, large bilobed cell, reedsternberg cells, reed sternberghodgkin , reedsternberg bcells, reed stenberg cell , hodgkin reed sternberg |
|  | PD-1 Inhibitors | PD-1 Inhibitors, Programmed Death Checkpoint Inhibitors, antipd, pd blockade, pd inhibitors |
|  | chimeric antigen receptors(CARs) | CARs , chimeric antigen receptors, car cells |
|  | granulocyte-colony stimulating factor | G-CSF, GCSF, granulocyte-colony stimulating factor |
| Site and region involvements | Cervical | neck, cervical, cervical lymph node, sides neck, neckdiagnosed |
|  | Chest/mediastinal | mediastinal, primary mediastinal, sternum, around heart, chest, mediastinal mass, around heart |
|  | Underarm | underarm, underarm lymph nodes, armpit, arm |
|  | Lymph node | lymph node, extranodal, primary lymph node site |
| Diagnosis and monitoring | Biopsy | biopsy, bone marrow biopsy, BMB |
|  | MRI or CT scan | Magnetic resonance imaging, CT scan, early mribased screening, fractal analysis application computed tomography |
|  | PET scan | FDG-PET scan, FDG-PET/CT, PET scan, PET-adapted de-escalation, PET-driven, Positron Emission Tomography, pet positive, pet negative, petnegative, negative pet, early petct scanning, petguided, pet metabolicscan, interim pet |
|  | X-rays | xray, x-ray |
| Sign and symptoms | Lymphadenopathy | enlarged lymph node, lump, lymphadenopathy, swollen lymph node, painless enl, swollen lymph nodes,nonpainful enlarged lymph nodes, lymph node swollen, mesenteric retroperitoneal lymphadenopathy |
|  | fever | fever, high fever, pelebstein fever, scratch fever, unexplained fever, high temperature, chills, fever night |
|  | sweats | night sweats, drenching night sweats, sweats |
|  | weight loss | unexplained weight loss, weight loss |
|  | fatigue/tiredness | CFS, cfsyndrome, chronic fatigue syndrome, fatigue, tiredness, extreme tiredness, lack of energy, no energy, extreme fatigue |
|  | cough and shortness of breath | Persistent cough, cough, persistent coughing, cough |
|  | abdominal pain and/or swelling | pain and swelling, abdominal pain, pain in stomach, swelling in stomach |
|  | Itchy skin | itching, pruritis, uncommon diffuse pruritis |
| Disease and conditions | Abnormal blood counts | Anemia, Low blood counts, Lymphocytic depletion, Monocytopenia, Neutropenia, Thrombocytopenia |
|  | Digestive diseases | Digestive problems, Acid reflux heartburn, Bloating, Constipation, Diarrhea, Digestive problems, Gastrointestinal disorders, Loose stools, Serious eating disorder, Ulcerative colitis |
|  | Cardiovascular diseases | Atherosclerosis, Cardiomyopathy, Heart damage, Heart disease, Heart disease, Ischemic heart disease, Longterm heart effects, Myocardial infarction, Pericarditis, Serious heart problems, Severe coronary artery disease, Stroke, Valve dysfunction |
|  | Pulmonary diseases | Acute lung toxicity, Asthma, Benign pneumoconiosis, Damaged lungs, Lung damage, Pneumonitis, Pulmonary edema, Pulmonary toxicity |
|  | Mental health conditions | Mental health, Anxiety, Depression |
|  | Secondary cancer | Acute myeloid leukemia, Aml, Basil cell carsinoma, Bladder cancer, Bone cancer, Breast cancer, Burkitts lymphomas, Colon cancer, Colon tumor, Diffuse large b cell lymphoma, Diffuse large bcell lymphoma, Glioblastoma, Hematologiccancer, Leukemia, Liver cancer, Lung cancer, Lymphomatoid papulosis, Mantle cell lymphoma, Melanoma, Mycosis fungoides, Myelodysplastic syndrome, Myelodysplastic syndromes, Nhl, Non-hodgkins lymphoma, Pancreatic cancer, Papillary thyroid carcinoma, Prostate cancer, Second cancer, Second malignancies, Skin cancer, Soft tissues cancer, Solid subsequent malig, Stomach cancer, Testicular cancer |
|  | Infectious diseases | Bacterial infection, Fungal infection, Herpes zoster, Hiv, Influenza, Leprosy, Pneumococcal pneumonia, Shingles |
|  | Thyroid disorders | Thyroid dysfunction, Injure the thyroid gland, Decreased thyroid function, Hypothyroidism, Hyperthyroidism |
|  | Hair loss | Hair loss, Short-term hair loss, Lost hair, Hair starting |
|  | Fertility issues | Azoospermia, Hypogonadotropic amenorrhea, Menstrual irregularities, Termination of ovarian function, Hypogonadotropic amenorrhea, Ovarian failure risk |
| Treatments | Chemotherapy | Chemo, Chemotherapy, A+AVD, AAVD, ABVD + rituximab, ABVD and BEACOPP, ABVD, ABVE PC, ABVE, Adriamycin- cyclophosphamide, Adriamycin PFS, Adriamycin RDF, Adriamycin, AVPC, AV-PC, BEACOPP, Bendamustine, Blenoxane, Bleo 15k, Bleomycin, Brentuximab vedotin + AVD, Carboplatin, Chlorambucil chemotherapy drug, CHOP + Rituximab, CHOP, Cisplatin, Combination doxorubicin, COPP, CVP + Rituximab, Cyclophosphamide, Cytarabine, Cytosar, Cytosar-u, Cytoxan lyophilized, Cytoxan, Dacarbazine, DHAP, Dose-escalated BEACOPP, Doxorubicin- cyclophosphamide, Doxorubicin, DTIC-dome, ESHAO, ESHAP, Etoposide, GDP, Gemcitabine, Gemzar, GVD, High dose cytarabine, High-dose Ara-C, High-dose cytarabine, ICE, IFEX, Ifosfamide, IGEV, Infugem, Liposomal doxorubicin, Matulane, Methotrexate, Navelbine, Neosar, OEPA-COPDAC, Oncovin, OPPA, OPPA/COPP, Paraplatin, Platinol, Platinol-AQ, Procarbazine, Rituximab ABVD, Salvage regimen, Tarabine PFS, Trexall, Velban, Vepemb, Vinblastine, VincasarPFS, Vincristine, Vinorelbine, Xatmep |
|  | Immunotherapy | Immunotherapy, Monoclonal antibody therapy, Targeted drug, Novel targeted therapies, Immune checkpoint agents, Adcetris, Adcetris firstline, Brentuximab, Brentuximab vedotin, Brentuximab vedotin adcetris treatment, Camidanlumab, Camidanlumab tesirine cami, Checkpoint inhibitor, Frontline brentuximab vedotin monotherapy combination, Keytruda, Monoclonal antibody, Nivolumab, Nivolumab treatment plan, Nivolumabinduced, Nivolumabs, Opdivo, Pembrolizumab, Riabni, Rituxan, Rituximab, Rubex, Ruxience, Tislelizumab, Truxima |
|  | Stem cell transplants | Transplant, Allogeneic bone marrow transplant, Allogeneic haematopoietic cell transplantation, Allogeneic stem cell transplant, Autograft, Autologous stem cell transplant, Bone marrow transplant, Haploidentical transplantation, Second autologous stem cell transplantation, Stem cell transplant |
|  | Radiation therapy | Radiotherapy, Radiationcancer drugs, Radiation treatment, Dimensional radiation therapy, Low dose ionizing radiation, 3D-CRT, Three-dimensional conformal radiation therapy, 4D-CT, Four-dimensional computed tomography, Image-guided radiation therapy, IGRT, Involved-site radiation therapy, ISRT, External beam therapy, Proton therapys, Chest IRR, Proton photon therapy |
|  | Treatment aid | Egg freezing, Gonadotropin-releasing hormone analogs, Ovarian freezing, Ppsv vaccine, Sperm banking, Sperm freezing |
