# Supplementary material for: Leveraging social media data to study disease and treatment characteristics of Hodgkin’s lymphoma Using Natural Language Processing methods
Source: PLOS Digit Health. 2025 Mar 19;4(3):e0000765. doi: 10.1371/journal.pdig.0000765 (PMC11922232; doi:10.1371/journal.pdig.0000765)
Supplement: S2 Table — (DOCX) [file pdig.0000765.s002.docx]

**S2 Table: List of words used to identify and exclude False Positive posts**

| "hodgkinjocelyn","campbells hodgkins","hodgkin guys","katehodgkin","hodgkinone","thodgkin petertimmins","dorothy mary hodgkins","howard hodgkinhis","hodgkin adam","hodgkinlift","hodgkincolored","hodgkin sacked","dorothy hodgkins","kerry hodgkinshades","goldmanhodgkinkatz","hodgkin college","merlin hodgkin","hopefully hodgkin","jordanhodgkin","hodgkin park","dorothy crowfoot hodgkin","hodgkinmolded","hodgkinwatercolor","eliot hodgkin","scotthodgkin","jonathan hodgkin","upulie hodgkin","rustyhodgkin","hodgkinlinen","adam hodgkin","dorothy hodgkin","riphowardhodgkin","hodgkin indian","hodgkin boss","emily hodgkin","hodgkin angela","george hodgkin","howardhodgkinimage","hodgkinthermography","rapp hodgkin","art hodgkins","hodgkins meet","solid hodgkin","jackhodgkin","lloyd hodgkin","howardhodgkin","toupees hodgkin","arthodgkins","mshodgkins","liz hodgkin","npg hodgkin","gt hodgkin","paul hodgkin","hodgkinsruby","hodgkin rocking","hodgkin dale","deehodgkin","john hodgkin","hodgkins collection","thodgkin lewisgoodall","thodgkin","hodgkin dexter","woy hodgkin","hodgkinscreenprints","not hodgkin","hodgkin books","samhodgkin","hodgkin chancellor","hodgkin replied","hodgkins nobel","elizabeth hodgkin","hodgkinomfrs","hodgkin told","hodgkin edward","eva hodgkin","hodgkin pickles","hodgkin building","phil hodgkin","niklaushodgkin","clive hodgkin","hodgkin blue","hopperhodgkins","mallory hodgkin","mrs hodgkin","hodgkin paintings","keenan hodgkin","joanna hodgkin","portrait hodgkin","davidcolquhoun hodgkin","hodgkin huxley","kate hodgkin","shite hodgkin","dislike hodgkins","howard hodgkin","khodgkin kierathompson","markhodgkin","keenanhodgkin","hodgkin franklin","thomas hodgkin","adamhodgkin","hodgkinhuxley","dorothyhodgkin","friederike thodgkin","howard hodgkins","hodgkinwood","roy hodgkin","dorothy mary hodgkin","hodgkina charcoal","sitarganj hodgkin","leonsquire thodgkin","hodgkintracy","corners hodgkin","hodgkinpainted","ryanhodgkin","maddiehodgkin","hodgkins part","hodgkins jiannecarlos","hodgkins raise","emilyhodgkin","hodgkinaquatint","helena hodgkin","made hodgkin female","hodgkin telling","brady hodgkin","weigh hodgkin","emhodgkin","hodgkin huxleys","hodgkin would","hodgkinnew","matthodgkin","thomas hodgkin william","hodgkin mullen","cureforcourtney" |
| --- |
